# NICE and NHS England leads the way to improve diabetes care with access to continuous glucose monitoring for people with type 1 diabetes

**DOI:** 10.1186/s12916-023-03014-2

**Published:** 2023-08-08

**Authors:** Sze May Ng

**Affiliations:** 1 Paediatric Department, Mersey and West Lancashire Teaching Hospitals NHS Trust, Ormskirk, L39 2AZ UK; 2 Department of Women’s and Children’s Health, University of Liverpool, Liverpool, UK; 3 Faculty of Health, Social Care & Medicine, Edge Hill University, Ormskirk, UK

**Keywords:** Continuous glucose monitoring, Type 1 diabetes, NICE, Diabetes technology

## Background

Type 1 diabetes care (T1D) management is complex and there is clear evidence that access to diabetes technologies has the potential to improve glycaemic management, quality of life and to prevent long-term diabetes complications. Continuous glucose monitoring (CGM) to date has been shown to improve the management of T1D. Real-time continuous glucose monitoring (rtCGM) and intermittently scanned glucose monitoring systems (isCGM) are new diabetes technologies that use a device inserted subcutaneously to measure interstitial glucose levels rather than capillary blood glucose from conventional finger pricks. While rtCGM provides a continuous real-time display of interstitial glucose, isCGM only displays the interstitial glucose level when scanned over the device. The National Institute for Health and Care Excellence (NICE) guidelines provides guidance on the quality of healthcare. Therefore, this commentary discusses new NICE guidance and NHS initiatives to widen access of CGM to improve diabetes care.

### NICE guidance recommends wider access to diabetes technologies and personalised approaches

Publication of new guidance by NICE (NG18) for children and young people with type 1 and type 2 diabetes was finalised in June 2022 and for adults (NG17) with T1D in August 2022 [1, 2]. NICE in the new updated guidance recommends that all children and adults with type 1 diabetes be offered rtCGM or isCGM based on their individual preferences, needs, characteristics, and the functionality of the devices available. NICE states that rtCGM or isCGM should be provided by a team with expertise in its use, as part of supporting people to self-manage their diabetes and that commissioners, providers and healthcare professionals should address inequalities in CGM access and uptake [1, 2]. These new recommendations from NICE 2022 represent a significant shift in providing universal health coverage and access to CGM for all people with type 1 diabetes and were adopted by NHS England as a commissioning body for the NHS. Previous NICE guidelines in both children and adults with type 1 diabetes had stringent criteria on who would be able to access CGM in the NHS. These new NICE guidances have significant implications as previous jurisprudence had ruled that NICE guidelines are important in determining the legal standards of healthcare [3].

### National Paediatric Diabetes Audit reports widening health inequalities

The National Paediatric Diabetes Audit (NPDA) 2021 reported the lowest use rt-CGM systems among children and young people of black ethnicity (11.7%), while children of white ethnicity had the highest use of these rtCGM (20.2%). Among the areas of deprivation, 14% of children from the most deprived were using rt-CGM compared to 25.2% from least deprived areas and children from ethnic minority communities have higher HbA1c compared to white children [4, 5], The NPDA further reported an increasing trend of widening health inequalities in the past 6 years. To date, there is limited research into the reasons for health disparities and worsening access to diabetes technology. A national survey undertaken by the UK Association of Children’s Diabetes Clinicians further reported that regional variations in access to rtCGM and isCGM for children and young people were prevalent, and also included variations in prescribing practice. Furthermore, funding of rtCGM and isCGM was largely dependent on commissioning groups within regions [6].

### CGM and hybrid closed loops improve outcomes

A recent study from the ALERT1 trial in adults found that glycaemic control using rtCGM with alert functionality provides more optimal results than those using isCGM without alerts [7]. Hybrid closed-loop systems require an rtCGM, an insulin pump and an algorithm to automatically adjust the amount of insulin delivered continuously. While still requiring adjusted manual meal-time insulin boluses, these systems help to maintain blood glucose within target ranges [8]. In 2022, NHS England’s hybrid closed loop study in children and adults became the first country-wide pilot initiative and the largest real-world study which provided universal health coverage of hybrid-closed loop systems. The children’s arm of the study reported a sustained improvement in glycaemic control, time-in-range and quality of life measures such as fear and worry of hypoglycaemia and improved sleep, in patients and their carers at 6 months after usage [9]. NHS England’s closed loop report provided further evidence for the next phase of the NICE Technology Appraisal TA 10845 guideline development of ‘Hybrid closed loop systems for managing blood glucose levels in type 1 diabetes’ that is currently under consultation [10].

These NICE recommendations represent a positive and significant shift in improving access to rtCGM and isCGM, whereas the previous NICE guidelines in both children and adults with type 1 diabetes had stipulated that CGM would only be accessible if certain stringent criteria were met. It is known that since the release of the guidelines in 2022, implementation has not yet occurred in many areas and clinical teams are advised to start early discussions with their local clinical commissioning groups. Successful technology adoption will reduce the health inequalities so evident in the national audits and will lead to a long-term impact on better diabetes health outcomes.

## Conclusions

NICE is responsible for robust evidence-based assessments of medicines, products, and technologies to see if they are beneficial and cost-effective in the NHS. NICE is also asked to consider the clinical effectiveness, safety and cost-effectiveness of their recommendations compared with current diabetes management options. Recent recommendations for CGM access and the forthcoming recommendations in the pipeline for hybrid closed loops will have the potential to transform the lives of people living with type 1 diabetes in the United Kingdom as an option to help them better manage their condition and improve their quality of life.

## Abbreviations

NHS: National Health Service
NICE: National Institute for Health and Care Excellence
NPDA: National Paediatric Diabetes Audit
isCGM: Intermittently scanned glucose monitoring
rtCGM: Real-time continuous glucose monitoring
T1D: Type 1 diabetes
